# Connexin Hemichannel Mimetic Peptide Attenuates Cortical Interneuron Loss and Perineuronal Net Disruption Following Cerebral Ischemia in Near-Term Fetal Sheep

**DOI:** 10.3390/ijms21186475

**Published:** 2020-09-04

**Authors:** Panzao Yang, Joanne O. Davidson, Tania M. Fowke, Robert Galinsky, Guido Wassink, Rashika N. Karunasinghe, Jaya D. Prasad, Sumudu Ranasinghe, Colin R. Green, Laura Bennet, Alistair J. Gunn, Justin M. Dean

**Affiliations:** 1 Department of Physiology, Faculty of Medical and Health Sciences, University of Auckland, Auckland 1145, New Zealand; p.yang@auckland.ac.nz (P.Y.); joanne.davidson@auckland.ac.nz (J.O.D.); t.fowke@mimetas.com (T.M.F.); g.wassink@auckland.ac.nz (G.W.); rashika.karunasinghe@auckland.ac.nz (R.N.K.); j.prasad@auckland.ac.nz (J.D.P.); s.ranasinghe@auckland.ac.nz (S.R.); l.bennet@auckland.ac.nz (L.B.); aj.gunn@auckland.ac.nz (A.J.G.); 2 The Ritchie Centre, Hudson Institute of Medical Research, Melbourne, Victoria 3168, Australia; robert.galinsky@hudson.org.au; 3 Department of Ophthalmology, Faculty of Medical and Health Sciences, University of Auckland, Auckland 1145, New Zealand; c.green@auckland.ac.nz

**Keywords:** interneuron, perineuronal net, hypoxia-ischemia, fetus, connexin hemichannel

## Abstract

Perinatal hypoxia-ischemia is associated with disruption of cortical gamma-aminobutyric acid (GABA)ergic interneurons and their surrounding perineuronal nets, which may contribute to persisting neurological deficits. Blockade of connexin43 hemichannels using a mimetic peptide can alleviate seizures and injury after hypoxia-ischemia. In this study, we tested the hypothesis that connexin43 hemichannel blockade improves the integrity of cortical interneurons and perineuronal nets. Term-equivalent fetal sheep received 30 min of bilateral carotid artery occlusion, recovery for 90 min, followed by a 25-h intracerebroventricular infusion of vehicle or a mimetic peptide that blocks connexin hemichannels or by a sham ischemia + vehicle infusion. Brain tissues were stained for interneuronal markers or perineuronal nets. Cerebral ischemia was associated with loss of cortical interneurons and perineuronal nets. The mimetic peptide infusion reduced loss of glutamic acid decarboxylase-, calretinin-, and parvalbumin-expressing interneurons and perineuronal nets. The interneuron and perineuronal net densities were negatively correlated with total seizure burden after ischemia. These data suggest that the opening of connexin43 hemichannels after perinatal hypoxia-ischemia causes loss of cortical interneurons and perineuronal nets and that this exacerbates seizures. Connexin43 hemichannel blockade may be an effective strategy to attenuate seizures and may improve long-term neurological outcomes after perinatal hypoxia-ischemia.

## 1. Introduction

Perinatal hypoxic-ischemic encephalopathy remains a major cause of brain damage in term and near-term infants worldwide [1]. Gamma-aminobutyric acid (GABA)ergic interneurons are the main inhibitory neurons in the brain and integration of GABAergic interneurons into the cerebral cortex in late gestation plays an important role in normal cortical development and function [2,3]. Glutamate decarboxylase (GAD) is an enzyme that catalyzes decarboxylation of glutamate to form GABA and is expressed in all GABAergic interneurons, while parvalbumin^+^, calretinin^+^, and calbindin^+^ interneurons form the major GABAergic interneuron subtypes. Experimental and human studies show that cortical interneurons are critical for normal cognitive processes, including executive function, learning, memory, and intelligence [2,4,5,6]. Limited human studies have also shown altered interneuron function after neonatal brain injury, including reduced cortical interneuron migration, loss of cortical GABAergic interneurons, and reduced GABAergic signaling [4,7]. Further, interneuron dysfunction and unbalanced excitatory–inhibitory activity in the cortex have been shown in developmental neurological disorders, including cognitive disabilities, epilepsy, and autism [5,6,8]. Thus, disruption of cortical inhibitory interneuron circuits may contribute to disability after moderate to severe hypoxic-ischemic encephalopathy in term infants.

Perineuronal nets (PNNs) are mesh-like structures of the extracellular matrix (ECM) that envelop the cell bodies and proximal neurites of subpopulations of neurons, including interneurons in the cerebral cortex [9]. PNNs are composed of a backbone of hyaluronic acid polymer joined to chondroitin sulfate proteoglycans via link proteins and tenascins [10,11]. PNNs help control normal GABAergic interneuron physiology, including regulating the formation and stabilization of synapses, ref [12] providing physical protection to neurons, mediating signal transduction, and controlling neuronal activity [13]. Disruption of PNNs has also been reported in a range of neurological diseases such as schizophrenia, ref [14] Alzheimer’s disease, ref [15] and epilepsy [16]. Further, we recently reported loss of PNNs on cortical interneurons after hypoxia-ischemia (HI) in term-equivalent fetal sheep [17]. Thus, changes in cortical interneuron PNN expression may be an important component of interneuron dysfunction and neurological disability following neonatal brain injury. However, there is limited evidence for therapeutic strategies to prevent interneuron injury, including PNN disruption, in the developing brain.

Gap junctions are intercellular channels that allow the movement of small molecules and ions between cells. Functional gap junctions are formed by docking of two hemichannels between adjacent cells. Undocked hemichannels are in a closed state under normal physiological conditions [18]. Connexin43 is the predominant connexin hemichannel in the brain and is mainly expressed on astrocytes [19]. In humans, the expression of connexin43 is markedly upregulated after cerebral ischemia [20]. In near-term fetal sheep, we reported abnormal opening of connexin43 hemichannels in the brain after HI, while blockade of connexin43 hemichannel signaling using a mimetic peptide (peptide 5) reduced brain injury and improved neuronal function [21,22]. However, the impact of connexin43 hemichannel opening on cortical interneuron cell death and function, including loss of PNNs, after neonatal HI is unknown. Thus, in the present study, we tested the hypothesis that blockade of connexin43 hemichannel opening during early recovery from HI in fetal sheep at 0.85 of gestation, when the brain maturation is broadly similar to the term human infant, [23,24] would improve cortical GABAergic interneuron survival, prevent loss of PNNs, and improve neurophysiological function.

## 2. Results

### 2.1. Survival of GABAergic Interneurons in the Parasagittal Cortex after Cerebral Ischemia

**Figure 1:**
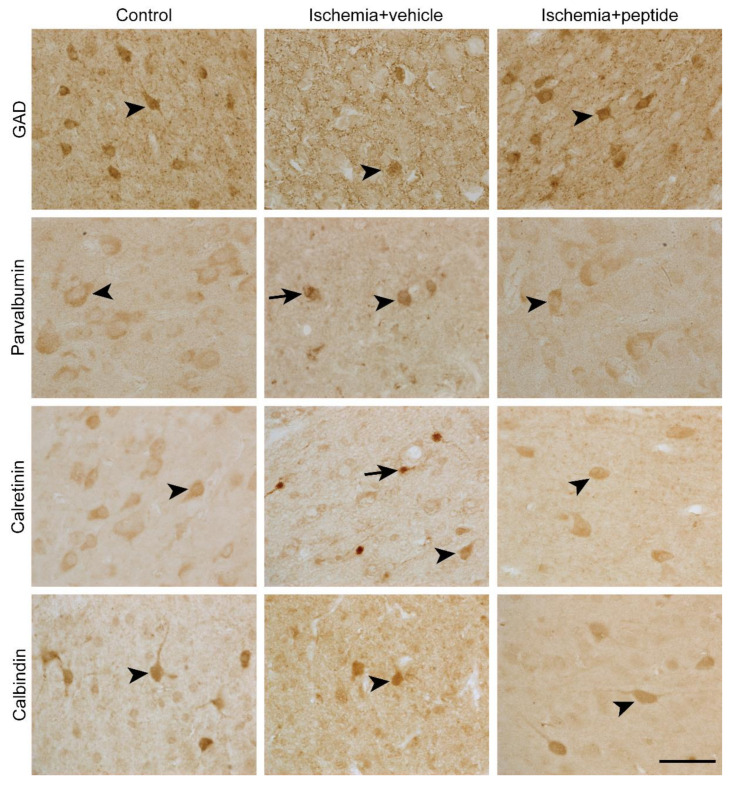
Photomicrographs of glutamate decarboxylase (GAD)^+^, parvalbumin^+^, calretinin^+^, and calbindin^+^ cortical interneurons in sham control, cerebral ischemia (ischemia + vehicle), and cerebral ischemia + mimetic peptide treatment (ischemia + peptide) groups at 7 days recovery. Black arrowheads show examples of neurons that were included in cell counting. Black arrows show examples of neurons with cytoplasmic aggregates, which were excluded from cell counting. Scale bar: 50 μm.

Representative photomicrographs of GAD^+^, parvalbumin^+^, calretinin^+^, and calbindin-28k^+^ neurons in all treatment groups are shown in Figure 1 (arrowheads). Note that ischemia + vehicle animals showed aggregates of dark parvalbumin and calretinin staining in the cytoplasm of some cortical neurons (arrows), which were not observed in sham control and ischemia + peptide animals.

**Figure 2:**
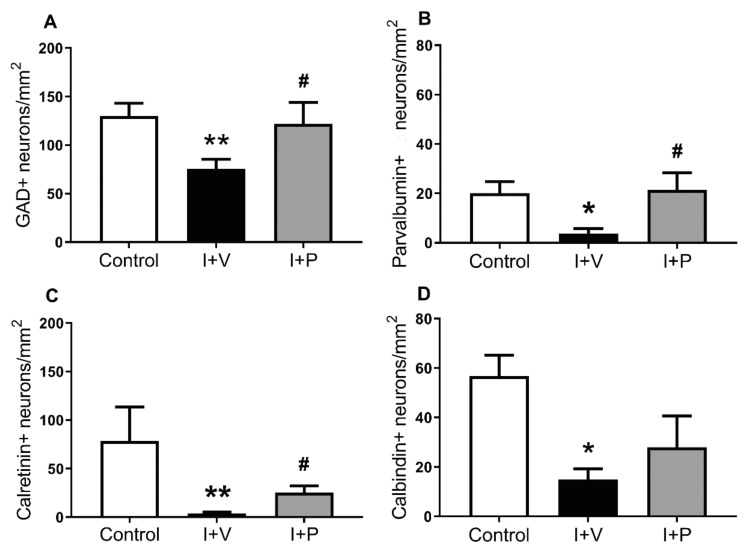
Interneuron survival in all layers of the parasagittal cortex in sham control (control), cerebral ischemia (ischemia + vehicle (I + V)), and cerebral ischemia + mimetic peptide treatment (ischemia + peptide (I + P)) groups at 7 days recovery. Densities (cells/mm^2^) of (**A**) glutamate decarboxylase (GAD)^+^, (**B**) parvalbumin^+^, (**C**) calretinin^+^, and (**D**) calbindin^+^ interneurons. Data are mean ± standard error of the mean. Sham control group (GAD, *n* = 8 animals; parvalbumin, *n* = 4; calretinin, *n* = 6; and calbindin, *n* = 5). I + V group (GAD, *n* = 10; parvalbumin, *n* = 6; calretinin, *n* = 6; and calbindin, *n* = 4). I + P group (GAD, *n* = 5; parvalbumin, *n* = 5; calretinin, *n* = 5; and calbindin, *n* = 5). * *p* < 0.05 and ** *p* < 0.01 ischemia vs. control; ^#^ *p* < 0.05 treatment vs. ischemia. Note that analysis of cell densities was performed on single antibody-labelled brain sections (two sections per marker per animal) visualized with 3,3′-diaminobenzidine tetrahydrochloride hydrate staining. Cells were counted in all layers of the first and second gyri of the parasagittal cortex (see Figure 6) using transmitted light microscopy (40× objective) and the fractionator probe (see Section 4. Materials and Methods, Image Analysis subsection). Positive cells were selected based on a typical pattern of cytoplasm staining and cellular morphology (c.f. control neurons in Figure 1).

Cerebral ischemia and peptide 5 treatment were associated with significant overall changes in interneuron density in the parasagittal cortex (Figure 2, one-way ANOVA; GAD^+^, *p* = 0.013; parvalbumin^+^, *p* = 0.03; calretinin^+^, *p* = 0.001; and calbindin^+^, *p* = 0.04). Post hoc analysis (Fisher’s Fisher’s least significant difference (LSD test)) showed a significant reduction in the density of GAD^+^ cells (*p* = 0.006; Figure 2A), parvalbumin^+^ cells (*p* = 0.03; Figure 2B), calretinin^+^ cells (*p* = 0.007; Figure 2C), and calbindin-28k^+^ cells (*p* = 0.04; Figure 2D) in the parasagittal cortex of ischemia + vehicle animals compared with sham controls. By contrast, in the ischemia + peptide group there was a significantly higher density of GAD^+^ cells (*p* = 0.036; Figure 2A), parvalbumin^+^ cells (*p* = 0.017; Figure 2B), and calretinin^+^ cells (*p* = 0.041; Figure 2C) but not of calbindin-28k^+^ cells (*p* > 0.999; Figure 2D), in the parasagittal cortex compared with ischemia + vehicle animals, indicating significantly greater survival of those cell types after peptide 5 treatment.

### 2.2. Changes in PNNs in Layer 6 of the Parasagittal Cortex Following Cerebral Ischemia

**Figure 3:**
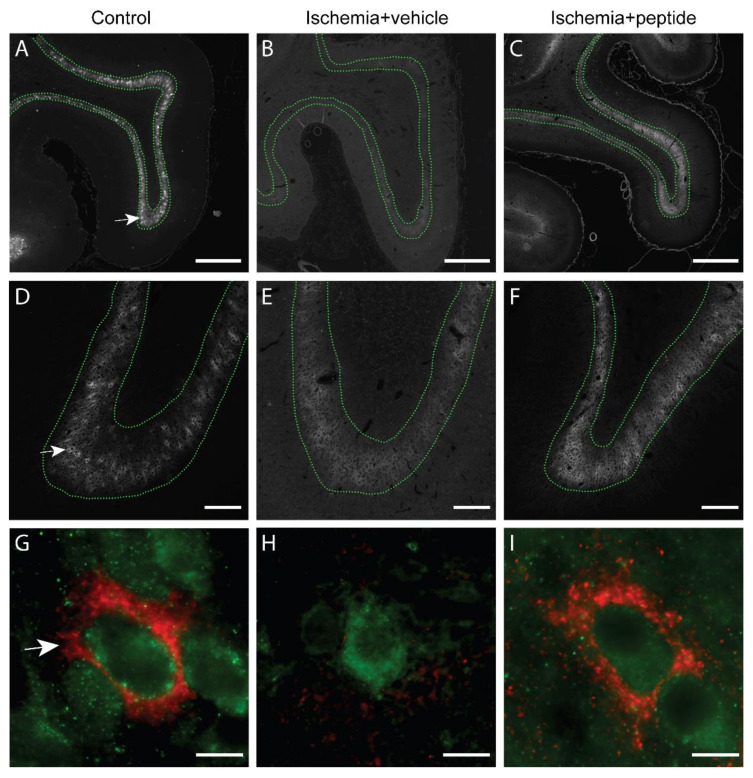
Immunofluorescent labelling of *Wisteria floribunda* agglutinin (WFA)^+^ perineuronal nets (PNNs) and glutamate decarboxylase (GAD)^+^ interneurons in the parasagittal cortex in the sham control (**A**,**D**,**G**), cerebral ischemia (ischemia + vehicle; **B**,**E**,**H**), and cerebral ischemia + mimetic peptide treatment (ischemia + peptide; **C**,**F**,**I**) groups at 7 days recovery. Note: images (**B** and **E**) (ischemia + vehicle group) were obtained at 5× higher exposure times than other images (sham control and ischemia + peptide groups) to allow visualization of WFA staining. (**A**–**F**) show WFA staining where the layer 6 region is defined within dotted lines. (**G**–**I**) show WFA^+^ PNNs (red) with GAD^+^ interneuron (green) staining. PNNs are indicated by white arrows (**A**,**D**,**G**). Scale bars: 1 mm (**A**–**C**); 200 μm (**D**–**F**); 10 μm (**G**–**I**).

Next, we assessed changes in PNN expression in layer 6 of the parasagittal cortex in the sham control, ischemia + vehicle, and ischemia + peptide groups. In uninjured control animals, *Wisteria floribunda* agglutinin (WFA) staining showed a pattern of dense pericellular labelling (i.e., PNNs; Figure 3A,D,G (arrows)), including on GAD^+^ cells (Figure 3G), and a more diffuse extracellular labelling (e.g., Figure 3D (layer 6 region defined within dotted lines)). Animals in the ischemia + vehicle group showed a marked reduction in WFA staining in layer 6 throughout the parasagittal cortex (Figure 3B) compared with controls (Figure 3A), which involved loss of both the PNN and diffuse ECM components (e.g., Figure 3E,H). By contrast, ischemia + peptide animals showed normal WFA labelling in both the PNN and diffuse ECM components (Figure 3C,F,I).

**Figure 4:**
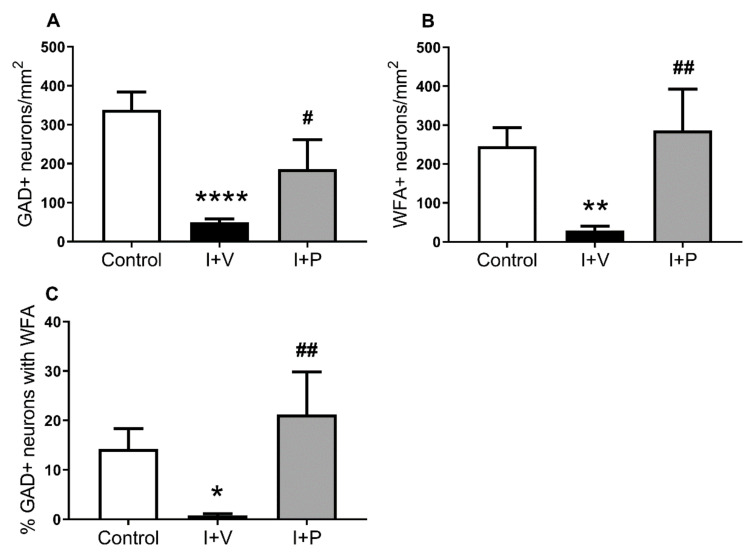
Interneuron survival and changes in perineuronal nets (PNNs) in layer 6 of the parasagittal cortex in sham control (control), cerebral ischemia (ischemia + vehicle (I + V)), and cerebral ischemia + memetic peptide treatment (ischemia + peptide (I + P)) groups at 7 days recovery. Densities (cells/mm^2^) of interneurons and PNNs identified with glutamate decarboxylase (GAD)^+^ (**A**), *Wisteria floribunda* agglutinin (WFA) (**B**), and the percentage of GAD^+^ interneurons with PNNs (**C**). Data are mean ± standard error of the mean. Control group (*n* = 6 animals). I + V group (*n* = 9). I + P group (*n* = 5). * *p* < 0.05, ** *p* < 0.01, and **** *p* < 0.0001 ischemia vs. control; ^#^ *p* < 0.05 and ^##^ *p* < 0.01 treatment vs. ischemia. Note that analysis of cell densities was performed on double-labelled brain sections visualized with immunofluorescence detection. GAD^+^ cells and PNNs (c.f. controls in Figure 3) were counted in layer 6 of the first and second gyri of the parasagittal cortex (see Figure 6) using reflected fluorescence microscopy (40× objective) and the fractionator probe (see Section 4. Materials and Methods, Image Analysis subsection).

**Figure 5:**
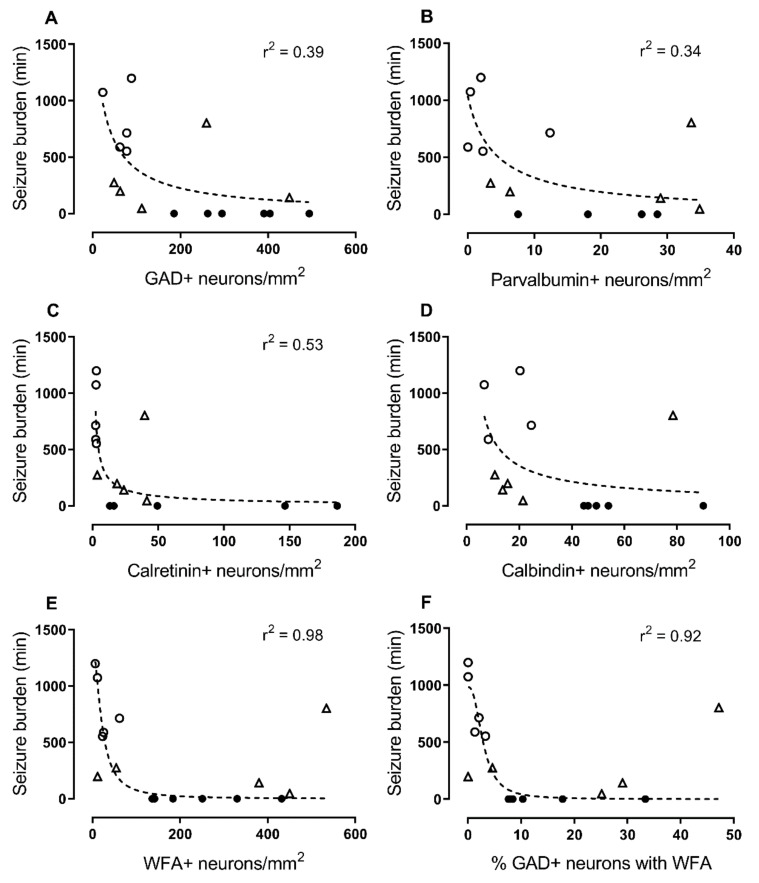
Nonlinear regression (sigmoidal curve fit) of the relationship of interneurons and perineuronal nets (PNNs) with total seizure burden (min): (**A**) Glutamate decarboxylase (GAD)^+^, (**B**) parvalbumin^+^, (**C**) calretinin^+^, (**D**) calbindin^+^ interneuron density, (**E**) *Wisteria floribunda* agglutinin (WFA)^+^ neuronal density (i.e., PNNs), and (**F**) the percentage of GAD^+^ neurons with PNNs (WFA^+^). Sham control group (*n* = 6 animals; closed circles). Ischemia + vehicle group (*n* = 5; open circles). Ischemia + peptide group (*n* = 5; open triangles).

We then quantified the effect of HI on the number of PNNs in layer 6 of the parasagittal cortex (Figure 4). There was a significant overall effect of treatment on the densities of WFA^+^ neurons (one-way ANOVA; *p* = 0.0041), GAD^+^ neurons (one-way ANOVA; *p* < 0.0002), and the percentage of GAD^+^ neurons expressing WFA (one-way ANOVA; *p* = 0.0085). Post hoc analysis (Fisher’s LSD test) showed a significant reduction in the density of WFA^+^ cells (*p* = 0.0069; Figure 5A), GAD^+^ cells (*p* < 0.0001; Figure 4B), and the percentage of GAD^+^ neurons expressing WFA (*p* = 0.0305; Figure 4C) in the parasagittal cortex of ischemia + vehicle animals compared with sham controls. By contrast, ischemia + peptide was associated with a significantly greater density of WFA^+^ cells (*p* = 0.003; Figure 4A), GAD^+^ cells (*p* = 0.03; Figure 4B), and the percentage of GAD^+^ neurons expressing WFA (*p* = 0.004; Figure 4C) in layer 6 of the parasagittal cortex compared with the ischemia + vehicle group.

### 2.3. Correlations with Seizure Burden

Finally, we examined the relationship between the density of GAD^+^ neurons or PNNs in cortical layer 6 with seizure burden (cumulative total min of all seizures) in all animals. There were negative relationships between seizure burden and the densities of GAD^+^ neurons (Figure 5A; *r*^2^ = 0.39), parvalbumin^+^ neurons (Figure 5B; *r*^2^ = 0.34), calretinin^+^ neurons (Figure 5C; *r*^2^ = 0.53), PNNs (Figure 5E; *r*^2^ = 0.98), and the percentage of GAD^+^ neurons with PNNs (Figure 5F; *r*^2^ = 0.92). Note that this relationship was strongest for PNNs and %GAD/PNNs. No relationship was found between seizure burden and the density of calbindin^+^ neurons (Figure 5D).

## 3. Discussion

There is increasing evidence that injury to cortical interneurons, including loss of PNNs and disruption of inhibitory interneuron circuits, is an important contributor to neurological disability following neonatal brain injury [17,25,26]. The present study demonstrates that blockade of connexin43 hemichannel opening with a mimetic peptide during early recovery from HI in term-equivalent fetal sheep improved survival of cortical GABAergic interneurons and prevented loss of cortical PNNs after 7 days of recovery. Further, this protection was associated with reduced total seizure burden measured from the cortical electroencephalogram (EEG). Overall, these data suggest that connexin43 hemichannel opening contributes to interneuron cell death and deficits in interneuron PNNs following HI, resulting in altered cortical inhibitory network function. Since peptide 5 at the levels used here does not uncouple gap junctions, [27] these data suggest that blockade of connexin hemichannels rather than gap junction communication between cells may be an effective treatment strategy for improving neurological outcomes following neonatal brain injury.

Damage to the parasagittal cortex in term infants after HI is highly associated with poor neurodevelopmental outcomes including cognitive and learning deficits and epilepsy [28,29,30,31]. In the present study, cerebral ischemia in near-term fetal sheep was associated with a pattern of parasagittal cortical damage after 7 days of recovery, which included a marked reduction in the survival of cortical GAD^+^, parvalbumin^+^, calretinin^+^, and calbindin^+^ interneuron populations. These findings are broadly consistent with previous studies showing loss and disrupted development of interneurons in the cerebral cortex and subcortical grey matter following HI in preterm and term fetal sheep [17,32,33,34,35] and neonatal rodents [36,37,38,39,40,41,42,43] and following ventilatory support in preterm baboons [44]. Although there are no comparable term human data, limited pathology and imaging studies in preterm infants have shown reductions in the numbers and complexity of cortical interneurons, refs [25,45] cortical interneuron migration, [7] interneuron neurogenesis, [46] and cortical GABAergic signaling [4]. Disruptions in interneuron function, resulting in reduced inhibitory control [47] and loss of the neurotrophic function of GABA, [48] are also implicated in many neurodevelopmental disorders including lissencephaly, intellectual disabilities, anxiety disorders, autism spectrum disorders, schizophrenia, and early life epilepsies [6,8,47,49,50,51,52,53]. Similarly, experimental disturbances in interneuron survival and function during brain development in animals are associated with a range of learning and behavioral deficits [54,55]. Thus, given the key roles of GABAergic neurons in normal cortical development and function, refs [3,56,57,58] loss of cortical neurons and associated disruptions in cortical interneuron circuitry may be important contributors to the long-lasting adverse neurophysiological outcomes associated with HI in term infants.

PNNs are also important contributors to inhibitory circuits [12,59,60,61]. In the present study, cerebral ischemia was associated with marked loss of the total number of PNNs and the percentage of PNNs on surviving GAD^+^ interneurons in layer 6 of the parasagittal cortex (the majority of PNNs are located within this layer in near-term fetal sheep [17]). Thus, although some of this PNN loss is related to interneuron death, these findings suggest that HI can directly affect the development and/or integrity of PNNs independent of interneuron survival. In support, disruptions in various components of PNNs were previously reported in the cortex and other subcortical structures following HI [62,63,64,65] or infection [25] in newborn rodents and after stroke or seizures in adult rats and sheep [16,66,67,68,69,70]. We also observed a strong relationship between loss of PNNs and seizure burden following HI, supporting a causative role of cortical interneuron dysfunction in the development of cortical hyperexcitability and seizures. Consistent with this, increased neuronal excitability and seizures were shown following degradation of PNNs in vitro [71,72] and following knockout or degradation of brain hyaluronan, the backbone component of PNNs, in neonatal and adult mice [73,74]. Interestingly, in experimental studies, loss of PNNs was reported to increase interneuron vulnerability to oxidative stress and cell death [75,76]. Given the established roles of oxidative stress in the evolution of neonatal brain injury, refs [7,77] loss of cortical PNNs may also contribute to the patterns of delayed interneuron cell death observed following neonatal HI. Collectively, these findings suggest that changes in cortical interneuron PNN expression may be an important component of interneuron dysfunction, seizure activity, and neurological disability following neonatal brain injury.

The exact mechanisms underlying the loss of interneurons and PNNs following term HI in the present study remain unclear. However, our findings of a marked reduction in interneuron cell death and PNN loss following treatment with a connexin43 hemichannel inhibitor from 90 min to 25 h after recovery from cerebral ischemia suggests a potential role for opening of hemichannels. There are 21 known connexin hemichannel isoforms, although connexin43 is the most highly expressed connexin in the brain and, as such, is a key connexin targeted for neuroprotection studies [78,79,80]. Abnormal opening of connexin hemichannels, including connexin43, following a range of CNS insults can result in spread of cellular injury through mechanisms involving loss of cellular membrane potential, abnormal calcium influx, and cell lysis as well as release of glutamate and cellular excitotoxicity [78,81,82,83]. Abnormal hemichannel opening is also associated with vascular disruption and hemorrhage [84,85] as well as inflammation, in particular inflammasome activation leading to the release of inflammatory cytokines in the CNS and other tissues [86,87,88,89,90]. Further, *N*-methyl-d-aspartate-induced cellular excitotoxicity is exacerbated in the presence of inflammatory cytokines and increased connexin43 hemichannel activity [91]. Thus, blockade of these pathogenic events with peptide 5 may have contributed to the survival of cortical interneurons after term HI in the present study [90].

Astrogliosis is a hallmark feature of perinatal brain injury [90,92,93,94] and reactive astrocytes play a key role in the evolution of brain injury [95,96,97,98]. Connexin43 is the predominant gap junction protein expressed on astrocytes [79,99,100] and the functions of astrocytes are controlled, at least in part, by connexin43 hemichannel signaling [18]. Critically, we and others reported that HI can trigger pathological opening of astrocytic connexin43 hemichannels, leading to reactive astrocytosis, altered astrocytic function, oligodendrocyte loss, interneuron injury, and seizures [101,102,103,104], while connexin43 hemichannel blockade using peptide 5 reduced these adverse effects [21,22,105,106,107].

With respect to the loss of PNNs observed following HI in the present study, under normal conditions, astrocytes play key physiological roles in the production and degradation of the ECM and PNNs via synthesis of proteoglycans and release of proteolytic enzymes [10,108,109,110,111]. However, changes in proteoglycan synthesis and excessive release of proteolytic enzymes by reactive astrocytes following injury can cause pathological ECM/PNN production/degradation [108,110,112,113,114,115]. For example, in preterm fetal sheep, HI was associated with increased hyaluronidase (degrades hyaluronan in ECM/PNNs) expression on reactive astrocytes from 1 day to 4 weeks recovery [116]. Similarly, rapid and persistent increases in expression of hyaluronidases, matrix metalloproteinases (e.g., MMP9; degrades chondroitin sulfate proteoglycans in ECM/PNNs [10,117,118]), and disintegrin and metalloproteinase with thromospondin motifs (degrade lecticans (e.g., aggrecan, brevican, versican, and neurocan) and phosphocan in ECM/PNNs [10,111,117,119]) with associated ECM disruption were reported following cerebral HI in neonatal [62,65,120,121] and adult [122,123,124,125,126] rodents. Comparable changes in various ECM proteases were reported in the plasma and/or brain following HI in human neonates [121,127] and stroke in human adults [128]. Further, experimental inhibition of ECM protease activity was reported to reduce neural injury following stroke in adult rodents [126,129]. Finally, in other tissues such as spinal cord injury, skin, and cornea of the eye wounds, connexin43 hemichannel opening is associated with development of extracellular matrix fibrosis [130,131,132]. Thus, speculatively, pathological connexin43 hemichannel-mediated astrocytosis after perinatal HI may result in aberrant ECM remodeling in the brain, including altered development and/or degradation of cortical PNNs. Conversely, inhibiting astrocytosis via connexin43 hemichannel blockade (e.g., see [85,132,133,134,135]) may prevent this ECM remodeling. Future studies examining changes in astrocytic proteoglycan synthesis and release of proteolytic enzymes with connexin43 hemichannel blockade after perinatal HI are required to validate this hypothesis.

Of note, it is possible that hemichannels other than connexin43 may contribute to evolution of brain injury following HI in the present study. For example, neuronal connexin36 hemichannels were reported to release ATP in a model of spreading depression in cultured neurons [136]. Further, opening of neuronal pannexin 1 hemichannels following anoxia caused sustained depolarization and cell death in cultured hippocampal slices, [137,138] while blocking pannexin 1 hemichannels was neuroprotective in adult rodent stroke models [139,140]. However, to our knowledge, there are no studies investigating the role of other hemichannels during recovery from perinatal ischemic brain injury. Importantly, peptide 5 was designed against the extracellular loop2 sequence of connexin43 [27], a region key for control of channel function, while the specificity of peptide 5 to connexin43 over other connexin isoforms was recently validated [141]. Further, in vitro studies showed that peptide 5 selectively inhibits connexin hemichannels at concentrations as low as 5 μM, although high concentrations (e.g., 500 μM) can inhibit both hemichannels and gap junctions [27]. This is supported by in vivo studies showing that low doses of peptide 5 (such as that used in the present study) are protective [21] while high doses can exacerbate injury [32]. Similarly, cytoplasmic edema in spinal cords segments is reduced with concentrations of peptide 5 that block hemichannels but is not prevented at high peptide 5 concentrations, [27] indicating that the retention of cell-to-cell communication is necessary for full protective benefit of hemichannel blockade.

In conclusion, the present findings suggest that opening of connexin43 hemichannels on astrocytes may contribute to the loss of interneurons, to deficits in interneuron PNNs, and to altered cortical inhibitory function, including delayed onset of seizures. Thus, targeting connexin43 hemichannel opening and the resulting reactive astrocytosis, which is a hallmark of injury to the developing brain [92,93], may be a viable strategy to attenuate seizures, to prevent pathological ECM remodeling, and to improve cortical interneuron survival and function after HI in term born infants. Of note, we previously reported that connexin43 hemichannel blockade could also reduce loss of striatal interneurons following HI in term fetal sheep [22]. Further, a reduction in interneuron loss in the cortex with erythropoietin treatment, [142] hippocampus with therapeutic hypothermia, [42] and striatum with bone marrow-derived mesenchymal stem cells [36] was reported following HI in neonatal rats and in the striatum with nitric oxide inhibition [33] or therapeutic hypothermia [143] following HI in preterm fetal sheep. Future studies are required to determine the exact timing of interneuron loss, altered PNN development, and expression of ECM remodeling enzymes after HI to help optimize treatment strategies.

## 4. Materials and Methods

### 4.1. Animals and Surgery

All procedures were approved by the Animal Ethics Committee of the University of Auckland (approval number 001942; 14 August 2017) and were performed in accordance with the New Zealand Animal Welfare Act 1999 and the University of Auckland’s Code of Ethical Conduct for the use of animals for teaching and research, approved by the Ministry of Primary Industries, Government of New Zealand. This manuscript is compliant with the ARRIVE (Animal Research: Reporting of In Vivo Experiments) guidelines for reporting animal research [144].

Aseptic surgery was performed on time-mated Romney/Suffolk fetal sheep between 118–124 days of gestation (term is 145 days). Food but not water supply was stopped 18 h before surgery. Long acting oxytetracycline (20 mg/kg; Phoenix Pharm, Auckland, New Zealand) was administered intramuscularly to ewes at 30 min before surgery. Anesthesia was initiated by intravenous administration of propofol (5 mg/kg; AstraZeneca Ltd., Auckland, New Zealand) and maintained using 2–3% isoflurane in oxygen (Bomac Animal Health, Glendale, NSW, Australia). Ewes received intravenous infusion of isotonic saline at a rate of approximately 250 mL/h to maintain fluid balance. The degree of anesthesia, maternal heart rate, and respiration were monitored during surgery.

The fetus was exposed after a maternal midline abdominal incision and polyvinyl catheters were inserted into the right and left fetal brachial arteries and amniotic cavity to measure mean arterial blood pressure. Electrocardiographic electrodes (AS633-3SSF; Cooner Wire, Chatsworth, CA, USA) were sewn across the fetal chest to record fetal heart rate. The vertebral-occipital anastomoses were ligated and inflatable carotid occluder cuffs were fixed around both carotid arteries [145,146]. Two pairs of EEG electrodes were fixed on the dura over the parasagittal parietal cortex (10 mm and 20 mm anterior to bregma and 10 mm lateral) with cyanoacrylate glue. Two electrodes were sewn in the nuchal muscle and a reference electrode was sewn over the occiput to record fetal electromyographic activity. An intracerebroventricular catheter was inserted into the left lateral ventricle (6 mm anterior and 4 mm lateral to bregma) for infusion of the mimetic peptide. Antibiotics (80 mg gentamicin; Pharmacia and Upjohn, Rydalmere, NSW, Australia) were then administered into the amniotic sac before the uterus was closed. The maternal laparotomy skin incision received infiltration of 10 mL 0.5% bupivacaine plus adrenaline (AstraZeneca Ltd.) for local analgesia. The maternal long saphenous vein was catheterized to allow postoperative maternal treatment and euthanasia.

### 4.2. Postoperative Care

Sheep were kept in separate metabolic cages in a temperature-controlled room (16 ± 1 °C, humidity 50 ± 10%) with ad libitum access to food and water and a 12 h light/dark cycle. Ewes received daily intravenous administration of antibiotics (600 mg benzylpenicillin sodium and 80 mg gentamicin; Novartis Ltd., Auckland, New Zealand) for 4 days. The maternal catheter was maintained patent by daily flushing and the fetal catheters were maintained by continuous infusion of heparinized saline (20 U/mL at 0.15 mL/h).

### 4.3. Experimental Protocols

Fetuses were randomized to sham control (*n* = 8), cerebral ischemia (ischemia + vehicle; *n* = 10), and cerebral ischemia + mimetic peptide 5 treatment (ischemia + peptide; *n* = 5) groups. At 128 ± 1 days of gestation, cerebral ischemia was induced by reversible inflation of the bilateral carotid occluder cuffs for 30 min with saline. Successful occlusion was confirmed by a suppression of EEG activity within 30 s of inflation. Carotid artery occlusion was not performed on sham control animals.

At 90 min after the end of occlusion, animals in the ischemia + peptide group received an intracerebroventricular infusion of a mimetic peptide (Peptide 5; H-Val-Asp-Cys-Phe-Leu-Ser-Arg-Pro-Thr-Glu-Lys-Thr-OH; Auspep, Tullamarine, Australia) dissolved in artificial cerebrospinal fluid at 50 μmol/kg/h for 1 h and at 50 μmol/kg/24 h for the next 24 h (infusion rate of 1 mL/h). Peptide 5 interacts with the extracellular loop of connexin43 to block connexin43 hemichannel activity [27]. The sham control and ischemia + vehicle groups received vehicle infusion of artificial cerebrospinal fluid. Continuous fetal EEG recordings began 24 h before bilateral carotid artery occlusion and continued through the whole course of the experiment. The animals were euthanized at 7 days after HI and the brains were removed, weighed, and processed.

### 4.4. Tissue Preparation

Preparation of the fetal sheep brains was performed as previously described [17,21]. In brief, postmortem fetal sheep brains were perfusion fixed in 10% phosphate-buffered formalin and embedded in paraffin. Coronal sections (10-μm thick) were cut using a microtome (Leica Jung RM2035; Leica Microsystems, Auckland, New Zealand). Two adjacent sections at the level of the mid-striatum [147] were selected from each animal for each analysis.

### 4.5. Immunohistochemical Staining

Immunohistochemical staining was performed as previously reported [17]. All washes were performed for 3 × 5 min in 0.1 M phosphate buffered saline (PBS). All primary and secondary antibodies were diluted with 3% normal goat serum (NGS)/PBS unless otherwise stated. For brightfield immunohistochemistry, sections were de-paraffinized in 2 × 15 min xylene and then rehydrated in a series of ethanol solutions (100%, 90%, and 75%; 5 min each), followed by 3 × 5 min PBS washes. Antigen retrieval was performed in 10 mM citrate buffer using the Antigen 200 Retriever (Electron Microscopy Sciences, Emgrid, SA, Australia) and the sections were washed in 3 × 5 min PBS. Endogenous peroxidase activity was blocked by incubating the tissue sections in 1% hydrogen peroxide in methanol. Sections were then washed in 3 × 5 min PBS, blocked in 5% NGS/PBS for 1 h at room temperature, and incubated in primary antibodies (Table 1) overnight at 4 °C. After washing in 3 × 5 min PBS, sections were incubated with biotinylated goat anti-rabbit or goat anti-mouse secondary antibodies (Table 1) for 3 h at room temperature, washed in 3 × 5 min PBS, and then incubated in ExtrAvidin-Peroxidase (Sigma-Aldrich Co., Saint Louis, MO, USA) diluted in PBS for 2 h at room temperature. Sections were washed in 3 × 5 min PBS and incubated in 3,3′-diaminobenzidine tetrahydrochloride hydrate (DAB; Sigma-Aldrich Co.) solution to generate a brown DAB reaction product before the reaction was stopped by washing in distilled water. Sections were then dehydrated in a series of ethanol solutions (75%, 90%, and 100%; 5 min each), followed by 2 × 10 min xylene. Slides were then cover-slipped with DPX mounting media (Sigma-Aldrich Co.).

For fluorescent immunohistochemistry, sections were deparaffinized, rehydrated, antigen retrieved, and blocked with 5% NGS as described above. Sections were then incubated in 0.1% avidin/PBS and 0.1% biotin/PBS for 15 min each to block endogenous biotin. Next, sections were incubated with biotinylated WFA (labels the *N*-acetylgalactosamine of the glycoconjugates enriched in PNNs [148,149]), rabbit anti-GAD65/67, and mouse anti-NeuN antibodies (Table 1) for 3 nights at 4 °C, washed in 3 × 5 min PBS, and then incubated in streptavidin-conjugated IgG Alexa Fluor 594, goat anti-rabbit IgG Alexa Fluor 488, and goat anti-mouse IgG1 Alexa Fluor 647 secondary antibodies (Table 1) and Hoechst 33258 (1:10,000; Thermo Fisher Scientific, Waltham, MA, USA) for 2.5 h at room temperature. Slides were then washed and cover-slipped using Vectashield mounting medium (Vector Laboratories, Burlingame, CA, USA).

### 4.6. Image Analysis

**Figure 6:**
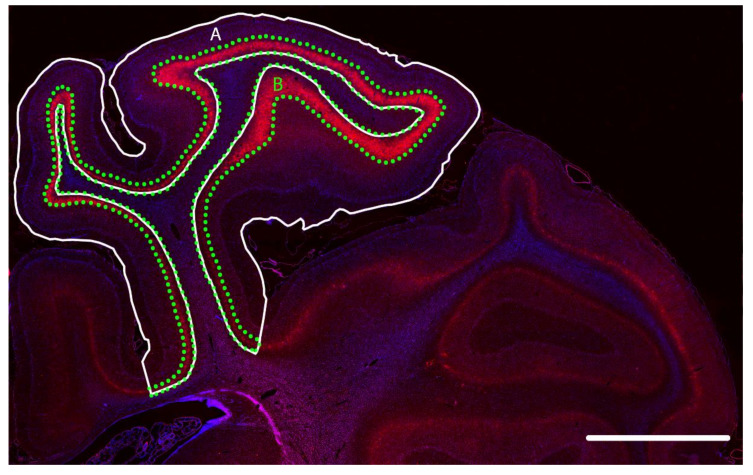
Sampling regions in the right parasagittal cortex of the near-term fetal sheep brain: Representative regions of interest for tracing (**A**) all layers of the cortices of the first two parasagittal gyri at the level of the mid-striatum (solid line) and (**B**) the dense *Wisteria floribunda* agglutinin (WFA; red staining)^+^ layer (layer 6; dotted line). Blue staining, nuclear labelling with Hoechst. Scale bar: 5 mm.

The methods for image analysis were previously described [17]. All quantification was performed by assessors (T.M.F. and P.Y.) blinded to the treatment groups. Imaging software (Stereo Investigator; MBF Bioscience, Williston, VT, USA) connected to a microscope (Zeiss AxioImager M2; Carl Zeiss Microscopy, LLC, Thornwood, NY, USA) equipped with a motorized stage (MAC 6000; MBF Bioscience) was used to trace the first and second parasagittal gyri of the right hemisphere of each brain section at 2.5× objective (Figure 6). The numbers of DAB-labelled GAD^+^, parvalbumin^+^, calretinin^+^, and calbindin^+^ neurons in all layers of the parasagittal cortex in both gyri were counted by transmitted light microscopy using the fractionator probe (40× objective; grid size: 500 × 500 μm; counting frame size: 150 × 150 μm; approximately 60 sites per gyrus). Positive cells were selected based on a typical pattern of cytoplasm staining and cellular morphology (c.f. control neurons in Figure 2) [22]. Note that cells with condensed cytoplasmic aggregates of GAD^+^, parvalbumin^+^, calretinin^+^, or calbindin^+^ staining (indicative of injured cells) were excluded from analysis. The neuronal densities (cell number/mm^2^) in the parasagittal cortex were calculated and two slides per animal were averaged to obtain the final data.

For quantification of fluorescently labelled WFA^+^ and GAD^+^ neurons by reflected fluorescence microscopy, the dense WFA immunoreactive layer (layer 6) in the parasagittal cortex was traced as described above (5× objective) using WFA, NeuN, and Hoechst labelling as guides (Figure 6), as we reported [17]. The numbers of WFA^+^, GAD^+^, and WFA^+^/GAD^+^ neurons in this layer were counted (40× objective; grid size: 500 × 500 μm; counting frame size: 100 × 100 μm; approximately 25 sites per gyrus) for each gyrus. The neuronal densities and percentage of WFA^+^/GAD^+^ neurons for each animal were calculated by averaging the density and percentage values of the two sections per animal.

### 4.7. Data Analysis

EEG data processing was performed using LabVIEW software (LabVIEW for Windows, National Instruments Inc., Austin, TX, USA). Seizures were recognized visually as sudden repetitive and evolving waveforms in the EEG signal lasting >10 s with an amplitude >20 μV [150]. Note that all physiological data from animals used in this study were previously reported [21,151]. The differences in neuronal cell densities between sham control, ischemia + vehicle, and ischemia + peptide groups were analyzed by one-way analysis of variance (ANOVA), followed by Fisher’s LSD test for post hoc analysis. Note that, for some animals, tissue sections for DAB staining of interneuron markers were not available because of technical reasons (see figure legends for specific animal numbers). The relationship between seizure burden and interneuron/PNN densities was examined by nonlinear regression with a sigmoidal curve fit and automatic outlier detection and elimination. *p* < 0.05 was considered statistically significant. All statistical analyses were performed using statistical software (GraphPad Prism v7.03; La Jolla, CA, USA). Data are presented as mean ± standard error of the mean.

## Figures and Tables

**Table 1:** Antibodies and markers used for immunohistochemistry.

Antibody	Dilution	Specificity	Source
**Primary Antibodies**			
Gamma-aminobutyric acid 65/67	1:200	GABAergic interneurons	Abcam, Melbourne, Australia
Parvalbumin	1:200	Parvalbumin interneurons	Swant Ltd., Marly, Switzerland
Calretinin	1:200	Calretinin interneurons	Swant Ltd.
Calbindin	1:200	Calbindin interneurons	Swant Ltd.
Biotinylated *Wisteria floribunda* agglutinin	1:400	Perineuronal nets	Sigma-Aldrich Co., Saint Louis, MO, USA
NeuN	1:20	Post-mitotic neurons	Merck Millipore, Billerica, MA, USA
**Secondary Antibodies**			
Biotinylated goat anti-rabbit IgG	1:200		Vector Laboratories, Burlingame, CA, USA
Biotinylated goat anti-mouse IgG	1:200		Vector Laboratories
Streptavidin-conjugated IgG Alexa Fluor 594	1:200		Thermo Fisher Scientific, Waltham, MA, USA
Goat anti-rabbit IgG Alexa Fluor 488	1:200		Thermo Fisher Scientific
Goat anti-mouse IgG1 Alexa Fluor 647	1:100		Thermo Fisher Scientific
